# Assisted Reproduction for a Same-Sex Couple: Interdisciplinary Preclinical Active Learning Module Combining Case-Based Small Group Discussion and Patient Panel

**DOI:** 10.1177/23821205241257325

**Published:** 2024-05-23

**Authors:** Xochitl A. Green, Kayla J. Flores Tindall, Ana L. Flores Tindall, Hana Anderson, Melody Y. Hou

**Affiliations:** 1 12218 University of California, Davis School of Medicine, Sacramento, CA, USA; 2 Department of Adult and Family Medicine, 23547 Kaiser Permanente Santa Rosa, Santa Rosa, CA, USA; 3 North Bay LGBTQI Families, Santa Rosa, CA, USA; 4 Department of Internal Medicine, Department of Cell Biology and Human Anatomy, University of California, Davis, Sacramento, CA, USA; 5 Department of Obstetrics and Gynecology, University of California, Davis, Sacramento, CA, USA

**Keywords:** Same-sex, lesbian, gay, bisexual, transgender, queer (LGBTQ), preclinical, assisted reproductive technologies, case-based small group discussion, patient panel

## Abstract

**OBJECTIVE:**

Physicians often feel they are not equipped to serve the lesbian, gay, bisexual, and queer (LGBTQ) community, but integrating education that incorporates LGBTQ content and perspective into an already-condensed medical school curriculum is challenging. We developed a preclinical active learning module on assisted reproductive technologies (ART) in LGBTQ care, integrating clinical and basic science content with patient perspective.

**METHODS:**

We created a module that combined a case-based small group discussion with a patient panel. We developed a case for discussion in collaboration with a female cis-gender same-sex couple who conceived through ART. A patient panel with the same couple followed the discussion. All first-year medical students attended both parts of the module. Prior to participation, students learned reproductive endocrinology and genetics concepts through lectures. After the module, students voluntarily completed an anonymous survey to evaluate self-perceived changes in familiarity and confidence with LGBTQ patients and satisfaction with the module.

**RESULTS:**

Of the 126 students who attended, 72 (57%) completed the survey. Of these, 69 (95.8%) felt the module gave them better perspectives on LGBTQ patient experiences, and 66 to 69 (92-96%) agreed the small group discussion achieved its learning objectives on LGBTQ health barriers and the application of ART. Students valued the patient panel (84.7%) and cited a better understanding of reproductive barriers for LGBTQ patients as its most valuable point.

**CONCLUSION:**

A preclerkship module combining a case-based small group discussion and patient panel on ART delivered in the context of a real-life LGBTQ patient experience provided an opportunity for the students to integrate basic science and clinical science knowledge to reflect on the healthcare needs of this patient population. Creating the case in collaboration with the same-sex couple and having them present their own experience provided an authentic perspective to students on reproductive healthcare issues and how they impact members of the LGBTQ community.

## Introduction

While the lesbian, gay, bisexual, and queer (LGBTQ) community makes up a growing acknowledged proportion of the United States (U.S.) population, inequities in their care remain or have worsened.^1–6^ Many medical students recognize that LGBTQ patients will account for a significant part of their future patient population, and understanding their healthcare needs is important to their roles as future physicians.^7–9^ Medical students who have received more exposure and training in LGBTQ patient care take better histories and have a greater knowledge of health care topics for the LGBTQ community.^10,11^ However, most medical students describe their LGBTQ curricula as “fair” or worse,^12^ and have at times instituted their own cocurricula to address this significant gap.^13^ The majority of published LGBTQ curricula content have an emphasis on improving attitudes or teaching LGBTQ-specific healthcare delivery.^8,10,11,14,15^ However, introducing new material that focuses on medical knowledge tailored to the LGBTQ population in the face of an increasingly condensed preclinical phase poses challenges and can lead to curricular imbalance and overload.^16,17^ To resolve this, integrating LGBTQ health content into a broader context such as reproductive endocrinology may provide a workable solution.^18^ Interweaving content on historically stigmatized patient populations such as those identifying as LGBTQ into basic science material during the preclinical phase not only enables earlier introduction of specific issues without sacrificing limited hours, but also cultivates comprehension of the relevance of key basic science concepts in medicine, regardless of patient identity.

A conscious effort to expand beyond content areas stereotypically associated with the LGBTQ population, such as HIV management and substance abuse, also helps to develop a broader outlook in medical students.^19–21^ Involving members of underserved communities in designing learning materials and/or facilitating sessions can address the curricular gaps, reduce implicit biases, and foster community engagement.^22,23^ One published LGBTQ learning module for preclinical medical students used a flipped classroom to teach genetics, reproductive endocrinology and LGBTQ healthcare, but the authors relied on a literature review, clinician and patient interviews to create their cases and did not incorporate the real-life experience of this patient population.^24^ One systematic review identified only 5 of 15 LGBTQ-related medical curricula that involved LGBTQ individuals in the creation or facilitation of these modules.^15,22,23^

To address the gap in LGBT content in the medical curriculum described above, we present a preclinical active learning module on assisted reproductive technologies (ART) for a female cis-gender same-sex couple. The module combined case-based small group discussions with a patient panel and was created through a collaboration between obstetrics and gynecology and genetics faculty and an LGBTQ-identifying student with her partner. By participating in the module, the students had the opportunity to apply their basic science knowledge in a real-life experience of members of the LGBTQ community.

## Methods

We conducted a survey study in 2020 of students at the University of California, Davis School of Medicine who underwent a preclinical active learning module consisting of 2 components, a case-based small group discussion and a patient panel, on ART for a same-sex female couple. The institutional review board at the University of California, Davis deemed this research as exempt (IRB identification. 1726827-1) and did not require separate informed consent documentation. Completion of the survey was considered as informed consent and this method of obtaining informed consent was approved by the institutional review board.

### Setting and participants

We delivered this module to the first-year medical students (*N* = 126) as part of an integrated preclinical course in which reproduction and genetics were taught concurrently at the University of California, Davis School of Medicine in Sacramento, CA in February 2020. Due to COVID-19-related restrictions, all learning activities evaluated here were delivered virtually and synchronously using Zoom (Zoom Video Communications, Inc., San Jose, CA). The module was designed as a combined case-based small group discussion and patient panel. Our goal was to provide a real patient case illustrating both the basic and clinical science concepts applied to ART and the types of barriers that prospective LGBTQ parents may experience, so students would reflect on and become cognizant of the circumstances surrounding the health care of this population. Prior to the module, students learned relevant concepts in reproductive endocrinology and genetics in lectures. These included: the menstrual cycle, early pregnancy maintenance, and genetic carrier screening, cytogenetics, mode of inheritance, prenatal testing, and genetic testing. To evaluate whether the module achieved its objectives, we distributed a voluntary survey to the participating students (described in the *Evaluation of the Module*).

Prior to the active learning module, the students took a 5-question quiz to assess their readiness for the module, which was standard for our curriculum. The questions were based on the materials covered in the corresponding reproduction and genetics lectures, and the students were allowed 3 attempts to achieve an 80% score that would be required to receive credit for attending the module.

### Development of the Case for Small-Group Discussion

The case was created in 2015 as a collaborative project between the obstetrics and gynecology director of the reproduction course and a now-practicing physician who was a student at our school at the time of case development. To develop the case, the student wrote an initial draft of her and her partner's experience of becoming parents through in vitro fertilization (IVF), with one partner carrying a pregnancy resulting from the other partner's egg and donor sperm. The focus on a female cis-gender same-sex couple would permit students to reason through what hormones would be necessary to induce one person to ovulate and to prepare another person to carry the resulting pregnancy. Session learning objectives were designed by an obstetrician-gynecologist, the genetics discipline director, and the student so that the students would apply the concepts of reproductive endocrinology and genetics they learned in the context of a female same-sex couple; these included the role and sequence of hormones involved in the menstrual cycle, early pregnancy and prenatal stages and genetic carrier screening for the prospective parents.

*Delivery of the Module* ^25^

The module was composed of case-based small group discussions facilitated by OBGYN clinical faculty and a patient panel with the graduated student and her partner. Attendance was required for both components.

We presented the 110-min case-based small group discussion component in 3 parts: (1) family building options, barriers to prospective parenthood, and expected healthcare maintenance for LGBTQ individuals; (2) preparation and induction of ovulation; and (3) IVF, embryo transfer, and early pregnancy maintenance. Each part included breakout sessions for students divided into small groups of 8 or fewer to discuss predetermined questions based on the case before gathering to present their groups’ answers as a larger cohort. For the 2020 module described here, the 8-student groups were assigned randomly via the Zoom breakout session algorithm. In this session we aimed to accomplish these main learning objectives: (1) discuss the importance of assessing reproductive goals and possible barriers for LGBTQ patients; (2) describe the workup to optimize pregnancy maintenance in patients who are pursuing IVF; (3) compare the roles and sequence of hormones important for ovulation and maintenance of pregnancy resulting from spontaneous fertilization versus IVF and (4) continue to develop interprofessional and communication skills relevant to LGBTQ care. After completion of the case-based session, students had access to the facilitator's guide for the small-group case discussions. The module's case is available as supplemental material.

Following the case-based small group discussion component, students attended a 50-min patient panel with the graduated student and her partner. They answered students’ questions about their experiences navigating the healthcare system as a same-sex couple seeking pregnancy in an open forum format. For the 2020 module described here, the panel was conducted over Zoom, with an instructor monitoring the chat function for questions to pose to the couple.

### Evaluation of the Module

We sought a convenience sampling of the students who participated in the module for analysis. Exclusion criteria included students who did not attend the module. We did not ask students to complete a preintervention survey. After the module, we sent weekly reminders to the students to complete a survey on their perceptions of the module until 6 weeks after the event, from February to March 2020. The survey, which was not a validated nor piloted survey due to the specific nature and learning objectives of this annual module, consisted of six 5-point Likert scale questions that evaluated learner satisfaction and reflection (Kirkpatrick Level 1).^26^ The survey asked whether students felt they achieved the following salient points represented by the learning objectives of the module: Q1. Recognize the barriers in reproductive care for LGBTQ patients; Q2. Explain the optimization of pregnancy initiated by IVF; Q3. Compare the role of different hormones for ovulation and pregnancy maintenance between spontaneous and IVF; Q4. Identify and explain the competencies in interpersonal communication and professionalism relevant to LGBTQ health care. Students were also asked whether they agreed with the following statements; Q5. Their understanding of the relevance and application of expanded carrier screening was improved; Q6. They obtained a better perspective of the experience of LGBTQ patients in the current health system. The students also voluntarily answered optional open-ended questions for narrative reflections.

### Data Analysis

We included data from all surveys, including incomplete surveys. We analyzed the Likert scale questions using descriptive statistics, calculating a proportion of each anchor, and reviewed the responses to the open-ended questions with a generic qualitative descriptive approach to extract and summarize the narrative reflections of the students, first through thematic analysis and then content analysis within a clinical competency framework.^27^ We used our school's graduation competencies, based on the 6 clinical competencies listed by the Association of American Medical Colleges and Accreditation Council of Graduate Medical Education: medical knowledge, patient care, interpersonal and communication skills, professionalism, systems-based practice, and life-long learning.^28–31^

## Results

**Figure 1.**
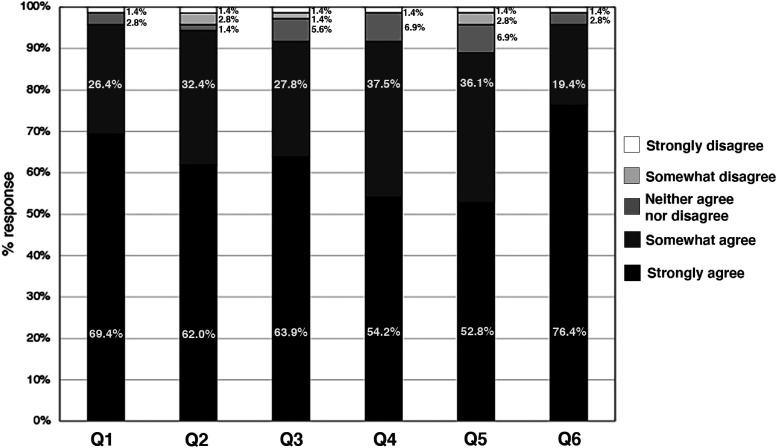
A summary of student feedback regarding an active learning module on assisted reproductive technologies for a female same-sex couple.

All 126 first-year medical students attended the required module composed of case-based small group discussions and patient panels as documented by Zoom attendance logs. Of those who attended the module, 72 (57%) responded to the survey. Most respondents strongly agreed or somewhat agreed that they achieved the intended learning objectives of the module: recognize the barriers in reproductive care for LGBTQ patients (*n* = 69/72, 95.8%); explain the optimization of pregnancy initiated by IVF (*n* = 67/71, 94.3%); compare the role of different hormones for ovulation and pregnancy maintenance between spontaneous and IVF (*n* = 66/72, 91.7%); identify and explain the competencies in interpersonal and communication and professionalism relevant to LGBTQ health care (*n* = 66/72; 91.7%). Students also strongly agreed or somewhat agreed that the module improved their understanding of expanded genetic carrier screening (*n* = 64 of 72, 88.9%) and gave them a better perspective of the experience of LGBTQ patients in our current healthcare system (*n* = 69/72; 95.8%) (Figure 1).

Out of the 126 students, 72 responded to 5-point Likert scale questions that asked whether the following learning objectives were fulfilled: Q1. Recognize the barriers in reproductive care for LGBTQ patients; Q2. Explain the optimization of pregnancy initiated by IVF; Q3. Compare the role of different hormones for ovulation and pregnancy maintenance between spontaneous and in vitro fertilization; Q4. Identify and explain the competencies in interpersonal and communication and professionalism relevant to LGBTQ health care. Students were also asked whether they agreed with the following statements: Q5. Their understanding of the relevance and application of expanded carrier screening was improved; Q6. They obtained a better perspective of the experience of LGBTQ patients in the current health system.

A total of 68 comments were obtained from the 33 (45.8%) students who answered at least one of the optional open-ended questions. When we grouped comments about the case-based small group discussion based on major themes we identified, we found that students considered the relevance of IVF to the LGBTQ community (13 of 33 comments, 39.3%) and learning about different ART approaches (8 of 33, 24.2%) their most important concepts learned from the discussion. Representative comments included: “It was important to learn the barriers to care and the legal barriers patients face with IVF/surrogacy”; “Some of the options for having a child I never knew before”; and “I don’t take for granted the importance of knowing each critical step in the process of fertilization, implantation, and pregnancy maintenance after of this [module].”

Students cited the patient panel component's most important concept learned was understanding the barriers regarding reproduction for the LGBTQ community (11 of 35, 31.4%). Representative comments included: “It was great seeing how they explain IVF to their children and the work they do to help other parents”; “It reinforced the need for us as emerging physicians to be knowledgeable beyond the cis/heteronormative standard of care”; and “I didn’t know that who gets to be the legal parent varies so much across states. It's bewildering that [someone] is a parent in California and may not be considered a parent in another state.” Students felt the patient panel was important to their learning (61 of 72, 84.7%). More students felt the panel should remain a mandatory component of the module for future classes (40 of 72, 55%) compared to 21 (29%) noting that the panel should be optional. Eleven (15%) did not answer the question.

When we grouped words and phrases within all the comments by clinical competencies,^30^ concepts that the students most frequently cited fell within the systems-based practice (32/68, 47.1%) and medical knowledge (16/68, 23.5%) domains (Table 1).

**Table 1.** Clinical competency categories^28–31^ of the student narratives regarding an active learning module on assisted reproductive technologies for a female same-sex couple. Students (*n* = 33) who responded to the open-ended survey questions about their most important concepts learned during the small group discussion and patient panel provided a total of 68 comments. Students may provide more than one comment and a single comment may count in more than one competency.

CLINICAL COMPETENCY	NO. COMMENTS (*N* = 68)	REPRESENTATIVE COMMENTS
Medical knowledge	16	“Reinforced my understanding of the basics: menstruation and pregnancy … because we are applying it to a family with 2 moms.” “The various ways to create a baby and to which extent science has been successful.” “I would like to learn more about the hormones being administered to both the egg donor and the person carrying the pregnancy, and the side effects of those injections.”
Patient care	13	“The need for us as emerging physicians to be knowledgeable beyond the cis/heteronormative standard of care.” “Physicians and healthcare workers are often not trained to provide fertility treatment to LGBT couples.”
Interpersonal and communication skills	7	“Never make assumptions and really engage my patients as they are often the source of the greatest knowledge with regards to their own health.” “Doctor understanding and support with appropriate communication are vital.”
Professionalism	7	“Don’t make assumptions about patients (or anyone else).” “Importance of care that considers the patients’ personal values, cultural beliefs, and desires.”
Systems-based practice	31	“The cost and legal barriers for LGBTQ communities when it comes to reproduction.” “In addition to being knowledgeable about medicine, it is important to be knowledgeable about laws that can affect our patients.”
Lifelong learning	0	

## Discussion

The majority of students expressed satisfaction that they achieved the learning objectives of this active learning module and found the session to be helpful for learning about ART and its relevance to the LGBTQ community. Narrative responses indicated that students valued the presentation of reproductive medicine in the context of LGBTQ patient care (Table 1). These findings show that our active learning module combining basic science instruction with LGBTQ healthcare can increase students’ self-assessed understanding of conception, early pregnancy maintenance, and LGBTQ care while maintaining curricular efficiency.^18^ Limitations of this study include its convenience sampling which is subject to nonresponse bias and the use of a nonvalidated or piloted survey due to the specific nature of the module. Another limitation of our study is that we evaluated student satisfaction and reaction, which is a Kirkpatrick Level 1 program evaluation and subject to recall bias, rather than comparing these postsession responses to a preintervention assessment to measure change. A pre- and posttest to assess improvement in knowledge would be the next step to assess the impact of this module, as well as a longer-term assessment of the persistence or fadeout of these effects.^32^ The low response rate to our open-ended questions is a limitation and likely due to conducting the survey in the course's final weeks and survey fatigue among other required student evaluations of the course and facilitators. Thus, the comments available may be subject to participation bias and not fully reflective of the larger cohort's perspectives on this module. The absence of comments within the life-long learning competency may be due to the lack of representation of this competency in the session's learning objectives. Adding additional explicit learning objectives regarding the role of physicians in improving their knowledge, skills, and attitudes with LGBTQ patients would help train students in this competency.^33^

Finally, our evaluation of this module occurred in 2020 during the COVID-19 pandemic, so this study's findings may not be generalizable to postpandemic in-person modules. Anecdotal feedback from discussion facilitators was that the use of breakout rooms on the Zoom platform during the pandemic made an assessment of discussion quality and providing feedback difficult compared to prior years’ in-person sessions, and we returned to in-person sessions as soon as this was deemed safe to do so the following academic year.

Recent iterations of the module expanded the patient panel to include a same-sex cis-gender male couple and their gestational carrier to allow for more diverse perspectives beyond the case, including discussion of more complex legal issues regarding the involvement of additional individuals who may become egg donors or gestational carriers. We want to emphasize that this module takes place in the context of an entire medical school curriculum, in which other concepts in LGBTQ care are further explored in other courses and clerkships. For example, this module may serve as a lead-in for instruction regarding attachment and psychological adjustment in LGBTQ families in pediatric, family medicine, or psychiatry clinical experiences.^34–36^ This case can also be modified for a patient who identifies with a gender minority and who may be pursuing gamete cryopreservation before, during, or after gender-affirming hormone therapy, or a patient who is pursuing IVF for the opportunity to use preimplantation genetic testing for hereditary disorders. Although this case is focused on LGBTQ+ family building with the goal of genetically related offspring, other forms of family building such as coparenting, adoption, foster care, child-free living, and other arrangements should also be recognized. For other medical schools seeking to adapt our module for their curriculum, we would recommend working with a same-sex couple, patients who identify as a gender minority, or other patients within the local community who have undergone ART to customize the case questions to their story, and have the patient(s) participate in a patient panel with students.^1–7^

## Conclusions

We developed an active learning module that integrated basic science teaching (genetics, early pregnancy mechanisms, embryology concepts) and clinical science knowledge in the context of an important issue in LGBTQ care that was well-received by students. Creating the case in collaboration with the same-sex couple and having them present their own experience provided an authentic perspective to students on reproductive healthcare issues and how they impact members of the LGBTQ community.

## Financial support

This research received no specific grant from any funding agency in the public, commercial, or not-for-profit sectors.

## Presentations

This project was presented in part at the American Congress of Obstetricians and Gynecologists District XIII and IX Annual District Meeting, Wailea Hawaii 29 September 2022.

## Supplemental Material

sj-docx-1-mde-10.1177_23821205241257325 - Supplemental material for Assisted Reproduction for a Same-Sex Couple: Interdisciplinary Preclinical Active Learning Module Combining Case-Based Small Group Discussion and Patient Panel Supplemental material, sj-docx-1-mde-10.1177_23821205241257325 for Assisted Reproduction for a Same-Sex Couple: Interdisciplinary Preclinical Active Learning Module Combining Case-Based Small Group Discussion and Patient Panel by Xochitl A. Green, Kayla J. Flores Tindall, Ana L. Flores Tindall, Hana Anderson and Melody Y. Hou in Journal of Medical Education and Curricular Development
