# Supplementary material for: Assisted Reproduction for a Same-Sex Couple: Interdisciplinary Preclinical Active Learning Module Combining Case-Based Small Group Discussion and Patient Panel
Source: J Med Educ Curric Dev. 2024 May 23;11:23821205241257325. doi: 10.1177/23821205241257325 (PMC11119406; doi:10.1177/23821205241257325)
Supplement: sj-docx-1-mde-10.1177_23821205241257325 - Supplemental material for Assisted Reproduction for a Same-Sex Couple: Interdisciplinary Preclinical Active Learning Module Combining Case-Based Small Group Discussion and Patient Panel [file sj-docx-1-mde-10.1177_23821205241257325.docx]

**Assisted Reproductive Technologies Case Faculty Guide**

**CASE DISCUSSION Assisted Reproductive Technologies**

Notes: Names have been changed to ficticious initials.

**USMLE Step 1 Relevance**

**Normal processes:** embryonic development, fetal maturation, and perinatal changes, including gametogenesis ; organ structure and function; pregnancy, including ovulation, fertilization, implantation; -cell/tissue structure and function, including hypothalamic-pituitary-gonadal axis, sex steroids, and gestational hormones

**Abnormal processes:** prenatal and perinatal counseling and screening

**Human development and genetics:** clinical genetics: testing, prenatal diagnosis, counseling

**Principles of therapeutics:** female reproductive tract, fertility drugs, gonadotropin-releasing hormone and gonadotropin replacement, including all gonadotropin releasing hormone antagonists, other therapeutic modalities affecting the reproductive system

**Gender, ethnic, and behavioral considerations affecting disease treatment and prevention, including psychosocial, cultural, occupational, and environmental:** emotional and behavioral factors, influence on person, family, and society, family planning and pregnancy, gender identity, sexual orientation

**Learning Objectives:**

- Discuss the importance of assessing reproductive goals and possible barriers for LGBTQ patients: *Interpersonal and Communication Skills, Information Sharing; Professionalism, Cultural Competence*
- Describe the workup to optimize pregnancy maintenance in patients who are pursuing IVF: *Patient Care, Diagnosis and Management*
- Compare the roles and sequence of hormones important for ovulation and maintenance of pregnancy resulting from spontaneous fertilization versus in vitro fertilization: *Knowledge, Fundamental Knowledge*

**Part 1**

A, a 26-year-old medical student assigned female at birth (AFAB, cisgender, she/her), and her partner B (AFAB, cisgender, she/her), also 26 years old, present to you interested in having a baby, and wondering where they should start.

1. What options do A and B have for expanding their family (including the different source combinations for egg, sperm, and uterus and how to put them together)?

- *Adoption, foster-care, co-parenting*
- *Home insemination – known or anonymous donor sperm*
- *Intrauterine insemination (IUI) - known or anonymous donor sperm, inserted by clinician*
- *Assisted reproductive technologies (ART)*
- *in vitro fertilization (IVF): ovum is removed, fertilized “in vitro” with sperm either by traditional IVF incubation or by intracytoplasmic sperm injection (ICSI), and then embryo is transferred into the uterus. If using ART, then the possible combinations are:*
  - *A carry pregnancy and use her ovum + donor sperm*
  - *A carry pregnancy and use B’s ovum + donor sperm*
  - *B carry pregnancy and use her own ovum + donor sperm*
  - *B carry pregnancy and use A’s ovum + donor sperm*
  - *Use donor ovum, donor sperm and use donor womb (gestational carrier/surrogate)*

*These options can also be offered to patients seeking single-parenting and patients who identify with a gender-minority. Care should be given to support patients who are seeking to build a family with genetically related offspring in this challenging process. Support should also be given to those patients who may decide not to pursue or continue this journey and choose to form their families in a different way.*

1. What are some common challenges faced by prospective LGBT parents in achieving their parenting goals in California? What if they were living in a different state?

*Students should try to go about finding legal advice for patients and laws in each state –* ***offer other states if students are from other states to look up, too, to help encourage discussion and to also show how hard it is to find this information****. California is generally considered a friendly state for LGBTQ family building with the recognition of pre-birth orders (a formal agreement between gestational carrier and intended parent(s) established prior to birth that protects the legal rights of the intended parents), but gestational surrogacy and pre-birth orders are not legalized across all states. In Texas, birth mother/surrogate is the mother of record regardless of genetic lineage. Even though same-sex marriages are recognized due to federal law, this may not abrogate individual state laws on adoption (such as second parent adoptions to establish a legal relationship between a genetically-unrelated parent and child) and fostering.*

*Beyond that, they should recognize stigma exists despite legality. Possible obstacles include lack of family support; overt and subtle discrimination by the foster care system, adoption agencies, reproductive endocrinology clinics, and labor and delivery units; financial cost of various parenting options; and legal barriers.*

Before telling them their options, you ask them if they have already thought about how they might want to go about it. They report they have thought about it extensively and would like to undergo IVF using B’s egg with donor sperm, with A carrying the pregnancy. You refer them to a reproductive endocrinologist, but they ask if there is anything they can do to start today. You explain they can begin by using combined hormonal contraceptives (CHCs) to synchronize their cycles.

1. Why would synchronizing their cycles be a helpful first step?

*Can be helpful to have A’s uterine lining to be in proliferative phase at same time as B’s, so ovum can be extracted from A, fertilized, and be placed immediately into a proper uterine environment which will support the pregnancy. If given to two people with uteruses and ovaries as the same time, a cycle of CHC hormones can allow their Day 1 of their following menstrual cycles to synchronize. For A and B, this will make it more likely that A’s uterus and B’s ovaries to be receptive to stimulation and implantation in the most optimal sequence. Faculty should highlight that this is an example of how contraception can be used for non-contraceptive indications such as cycle suppression, as well as to manage abnormal uterine bleeding, dysmenorrhea, ovarian cyst suppression, and acne or hirsutism.*

*Synchronizing their cycles however may not be necessary if the patient(s) elect to do frozen embryo transfer, in which embryos are frozen and thawed at a later time to match the recipient’s uterine preparation, either within their own cycle (“natural cycle IVF”) or traditional IVF with hormone treatments to prepare the uterus for embryo transfer as described below.*

1. While you have these two healthy 26-year-old women in your office, what are important health maintenance items to consider?

***Discuss how discrimination and assumptions about LGBT can prevent good health care maintenance, such as need for pap and STI screening and even contraception.*** *It is a common myth that women in same-sex relationships are not at risk for STIs, including HPV, In one study, 70% of lesbian-identifying women reported having had intercourse with a man in the past, and HPV can be transmitted via oral and manual stimulation. Patients who have cervices and are otherwise at risk for cancer of these organs are less likely to receive Pap screening when assumptions are made about their gender presentation or their sexuality. So make sure to do the following tests if they are at risk and they consent to screening:*

*Gonorrhea/Chlamydia screening; Pap screening with HPV testing as needed for cervical cancer; immunizations including influenza and HPV*

You refer them to an IVF center that specializes in reproductive technologies.

1. The IVF specialist asks that you have Baget an expanded carrier screening. What are the benefits and limitations of this test?

*Expanded carrier screening should be offered to all patients, although only 15% of obstetricians report offering this to all their patients and 52% offer to patients who request this. Insurance does not always cover this test, so cost to the patient should also be considered. This screens for a large number of conditions without regard to race or ethnicity. The majority of testd are for autosomal recessive disease, but some may test for X-linked and autosomal dominant single gene disorders. The specific tests on a laboratory panel vary by laboratory and may continue to expand over time as more disorders are added.* ***A benefit of this test would be that If B is shown to be a carrier for a specific disorder, the couple can choose a sperm donor that is not a carrier for the same disorder.*** *Limitations include that some of the disorders identified may have unclear clinical significance, result in only mild to moderate health complications, have variable phenotype, or have onset in adulthood that are easily detected or treated. A positive screening test does not necessarily mean that the fetus is affected but may also have implications for the patient and other family members. We also can only test for disorders we know, and so a negative screen does not necessarily guarantee a healthy fetus and child.*

1. The specialist asks you to obtain TSH, fasting glucose, and prolactin levels for A. Why do you think they are interested in these labs? Are these labs cost-effective?

*Discuss effects of endocrinopathies on pregnancy outcomes. Poorly controlled hyperglycemia, thyroid disease, and hyperprolactinemia are all associated with early pregnancy loss (EPL) and spontaneous abortions (SAB).* ***In a healthy young person with no previous history to suggest these disorders such as irregular menses or nipple discharge in the case of hyperprolactinemia, these may not be cost-effective, especially if they may have to pay out of pocket.*** *You might be able to have these covered by insurance if ordered as general health maintenance labs.*

1. The specialist also wants A to obtain a saline sonohysterogram (an ultrasound of the uterus after infusing it with saline, out of pocket cost approximately $800-1200). Why do you think this test was requested? Do you feel it’s necessary for A to have this test?

*Test can reveal uterine/mullerian anomalies such as septated uterus, or tubal anomalies such as tubal occlusion and scarring, intrauterine adhesions, and obstructive leiomyomas that may prevent implantation or cause pregnancy loss. However, A has not established a clinical diagnosis of infertility (historically defined as 6-12 months of penile-vaginal intercourse without contraception that does not result in pregnancy) and so this test may not be necessary prior to attempting conception. Further, although the American Society for Reproductive Medicine has redefined infertility to be inclusive of anyone needing donor gametes or embryos for a successful pregnancy, a patient’s insurance may not cover infertility-related care. Again, consider the financial constraints on your patient.*

**Part 2**

B and A present to the IVF specialists. After discussing their goals, they are ready to start the IVF process.

B starts on 3 subcutaneous injectable medications to stimulate the growth of her eggs: leuprolide, follitropin alpha, and menotropins.

1. What hormones might these medicines mimic, and what are the roles for these drugs in assisted reproductive technology (ART)?

***Leuprolide****=Gonadotropin Releasing Hormone Agonist (GnRH agonist) – when given, it continuously suppresses endogenous gonadotropins since physiologic GnRH is pulsatile (USMLE drug)*

***Follitropin****=Follicle Stimulating Hormone (FSH) – controls stimulation of follicular development*

***Menotropins****=FSH and LH activity - controls stimulation of follicular development*

*Combined, folliitropin (day 1) followed by menotropins (day 2-5) has excellent results.*

**Clinical Connection: Leuprolide is used in transgender pre-pubescent patients in order to delay puberty to give them more time to decide if they want to take gender-specific hormones to permanently develop their bodies to match their gender; it is similarly used to treat central precocious puberty.*

1. Two weeks have passed. B’s ovaries now have multiple large follicles, as shown below. What symptoms might B be feeling?
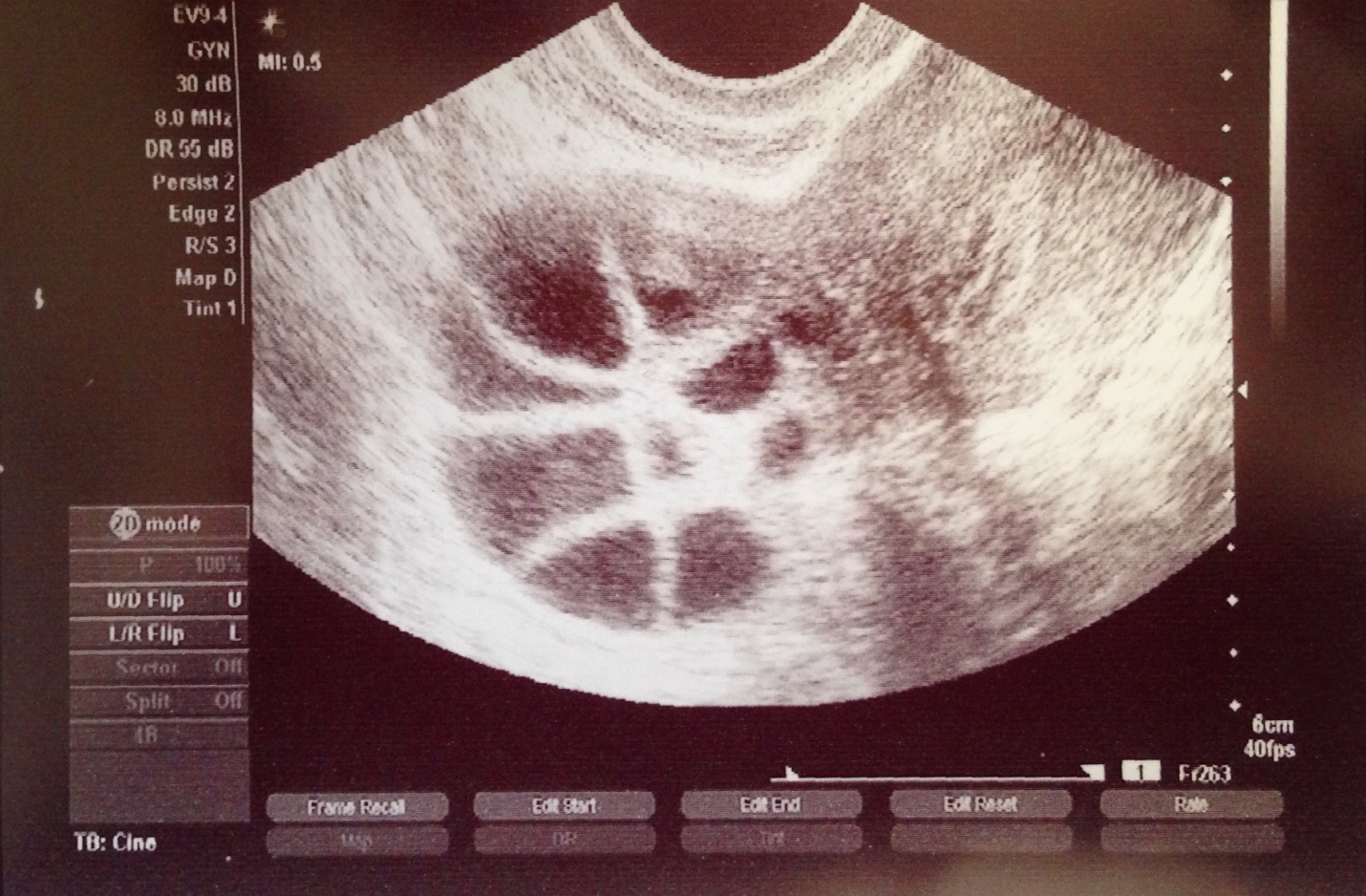


***Point out how big these ovaries are – these ovaries at 8 cm!*** *B hurt a lot during this time period.*

On “stimulation day,” a trigger injection of choriogonadotropin alfa is given to B, starting the clock after which eggs need to be retrieved at a specific time.

1. What natural hormonal process is the trigger injection imitating, and why is there a time limit?

*Choriogonadotropin alfa is a recombinant hCG injection; this administration mimics the LH surge which causes maturation of the eggs in preparation for ovulation; eggs need to be retrieved before ovulation occurs since once ovulation occurs, egg hunting becomes very difficult as they are released into the peritoneum!*

Twenty-three eggs are retrieved, 18 of which are mature. Each egg is bathed in thawed sperm in a petri dish, and 15 successfully fertilize. The infertility clinic generally requires intra-cytoplasmic sperm injection (ICSI) be performed for an additional $1500 when frozen sperm is being used.

1. What is involved with ICSI, and when would it be necessary? What fertilization processes are A and B hoping to preserve by not using ICSI?

*ICSI is when a single sperm is injected directly into a mature oocyte – it’s used when the sperm cannot penetrate the egg, sometimes a reason for male infertility. Usually, a successful spermatozoon can penetrate the cumulus oophorus to reach the zona pellucida of the oocyte, and then enters the oocyte via an acrosome reaction. This acrosomal reactions prompts the oocyte to complete meiosis 2 to become a haploid cell (and polar bodies), and block other spermatozoa from entering. The female and male pronuclei will fuse, creating a diploid single cell called a zygote.*

1. Meanwhile, A has been taking injectable hormones as well: leuprolide, estradiol, and progesterone. What hormonal sequence would be necessary for A’s preparation for pregnancy, and why?

***Leuprolide****=Gonadotropin Releasing Hormone Agonist (GnRH agonist) used continuously to block A from ovulating*

***Estradiol****=form of estrogen typically made by the ovaries, stimulates A’s endometrial proliferation to be receptive to the pregnancy, followed by*

***Progesterone*** *– maintains A’s endometrium to support implantation*

*Since we’re suppressing A’s endogenous LH and FSH with leuprolide, we need to replicate the* ***proliferative phase*** *and* ***secretory phase*** *of the uterus – estrogen, then progesterone. Sometimes this step is not necessary if the partners’ cycles are completely synced (which you tried to do with the CHC cycles earlier), but exogenous sequential hormone administration can help optimize the chances of synchronicity.*

The zygotes begin dividing over the next two days, each becoming a **morula** encased in its zona pellucida. On day 5, six of the embryos have become **blastocysts**.

The blastocyst pictured below is inserted into the fundus of A’s uterus using a thin straw inserted through the cervix.


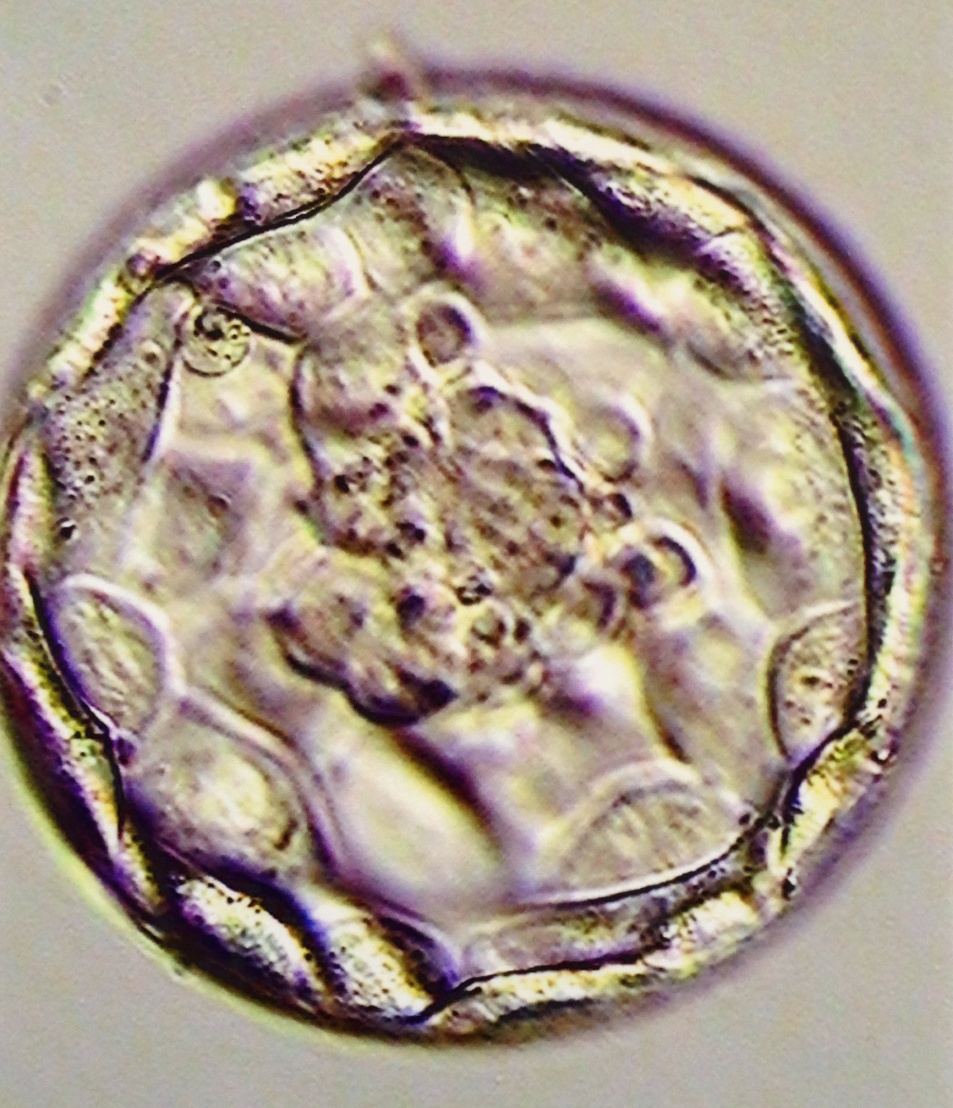


1. For the next 12 weeks, B will inject A with intramuscular progesterone injections. Why are these injections necessary? What happens if they miss doses?

*Usually, the corpus luteum releases progesterone to maintain the uterine lining until the placenta takes over, usually by 7 weeks although many specialists prefer to continue injections until 12 weeks. The withdrawal of progesterone could cause A to shed her uterine lining and lose the pregnancy; just as the withdrawal of progesterone when the corpus luteum dies causes a non-pregnant woman to menstruate.*

Three days later, A feels the slightest hint of nausea. Her hCG levels confirm she is pregnant, and the levels increase appropriately over the next several weeks, as shown in the pink dots on the graph below. A 6 week ultrasound shows an embryonic pole, gestational sac, and yolk sac, shown below.


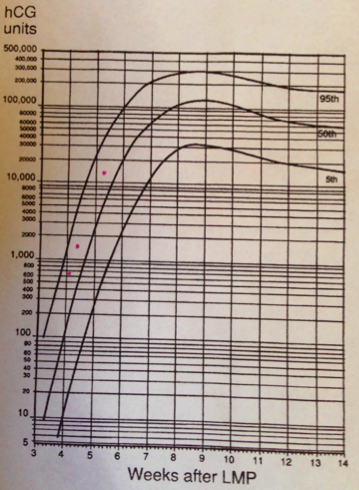


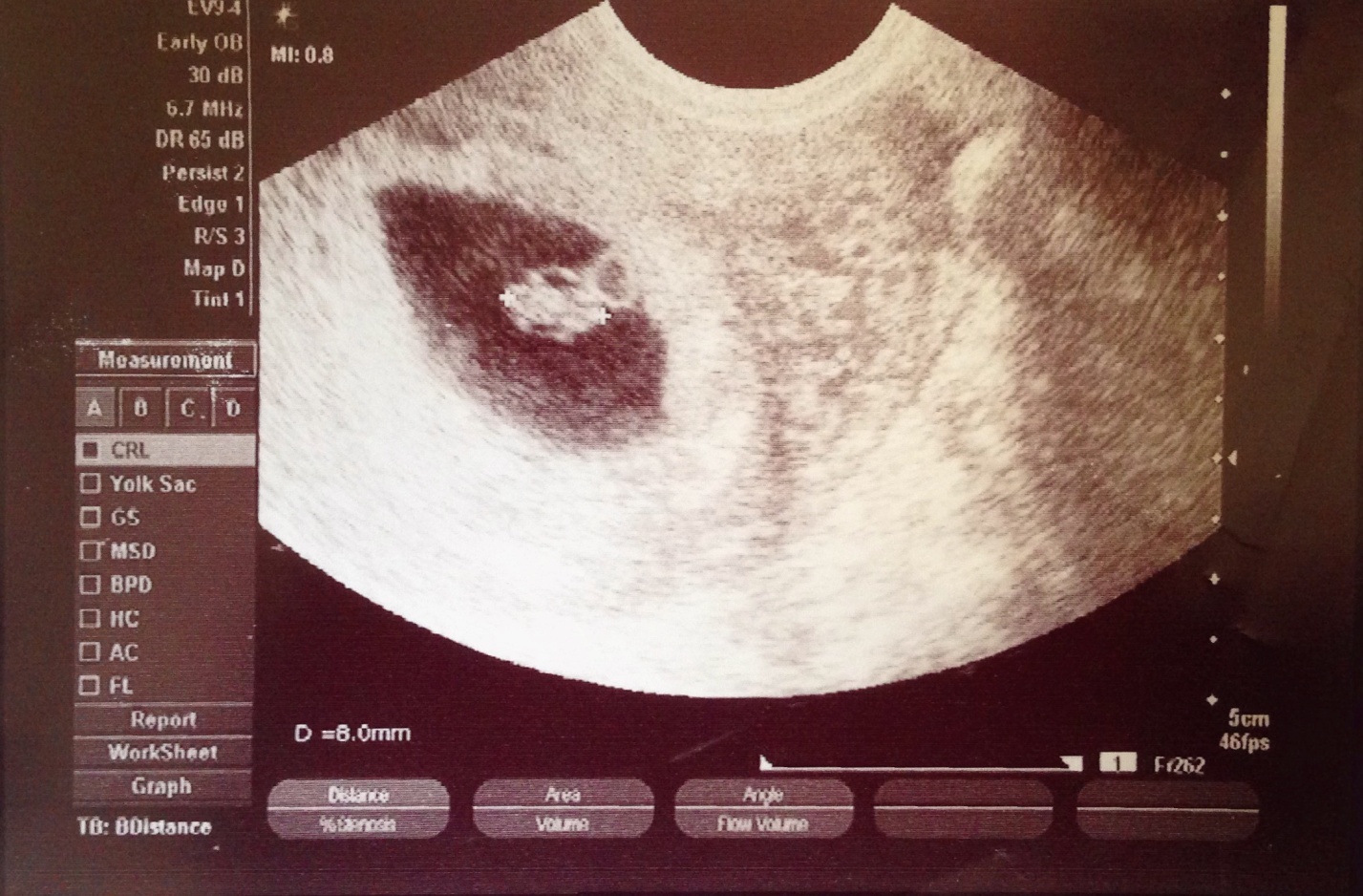


1. At 12 weeks, B stops injecting A with the progesterone shots. Why are the progesterone injections no longer necessary?

*Placenta has taken over production of progesterone. Progesterone is “PRO-GESTation” and serves to maintain the pregnancy by relaxing uterine smooth muscle, stimulating endometrial gland secretions and spiral artery development, inhibiting FSH and LH, and producing thick cervical mucus that inhibits sperm motility.*

The rest of the pregnancy and labor are uncomplicated.

**ENRG Reproduction – CASE DISCUSSION Pregnancy**

Helpful video on IVF: <https://www.youtube.com/watch?v=fy61qs_tXTQ>

**Take Home Points:**

- Leuprolide=Gonadotropin Releasing Hormone Agonist (GnRH agonist) – when given continuously🡪 suppresses endogenous gonadotropins; physiologic GnRH = pulsatile

- LH surge🡪maturation of the eggs in preparation for ovulation

- Estradiol=form of estrogen typically made by ovaries🡪stimulates endometrial proliferation in preparation for pregnancy

-Progesterone🡪maintains endometrium to support implantation (↓ progesterone🡪↓ fertility)

-hCG (from syncytiotrophoblast cells of fetus) can be detectable as soon as 9 days after conception when implantation completes, elevated hCG also seen in ectopic pregnancy, hydatidiform mole, ovarian germ cell tumors, choriocarcinoma, Down syndrome

-Oogenesis: 2^o^ haploid oocytes (2N) arrested in METaphase of meiosis II until fertilization occurs. Remember: “An egg MET a sperm.” (First Aid Mnemonic)

-Spermatozoon enters ovum via acrosomal reaction. Remember: “‘Gonium’ is going to be a sperm; ‘Zoon’ is ‘Zooming’ out of the cell.” (First Aid Mnemonic)

- Zygote = 1 cell, morula = solid ball of cells, blastocyst = hollow ball of cells, embryoblast = inner cells of blastocyst, embryo 🡪 fetus ~ 9-10 wks

| **Quad Screen** | **AFP** | **hCG** | **inhibin A** | **estriol** |
| --- | --- | --- | --- | --- |
| **Trisomy 21 (Down)** | **↓** | **↑** | **↑** | **↓** |
| **Trisomy 18 (Edwards’)** | **↓** | **↓** | **↓** | **↓** |

-Helpful Mnemonic: 2 values are DOWN in DOWN syndrome
